# Supplementary material for: Arabidopsis thaliana MYC2 and MYC3 Are Involved in Ethylene-Regulated Hypocotyl Growth as Negative Regulators
Source: Int J Mol Sci. 2024 Jul 23;25(15):8022. doi: 10.3390/ijms25158022 (PMC11311335; doi:10.3390/ijms25158022)
Supplement: Supplementary file 1 [file ijms-25-08022-s001.zip › ijms-3082008-supplementary.pdf]

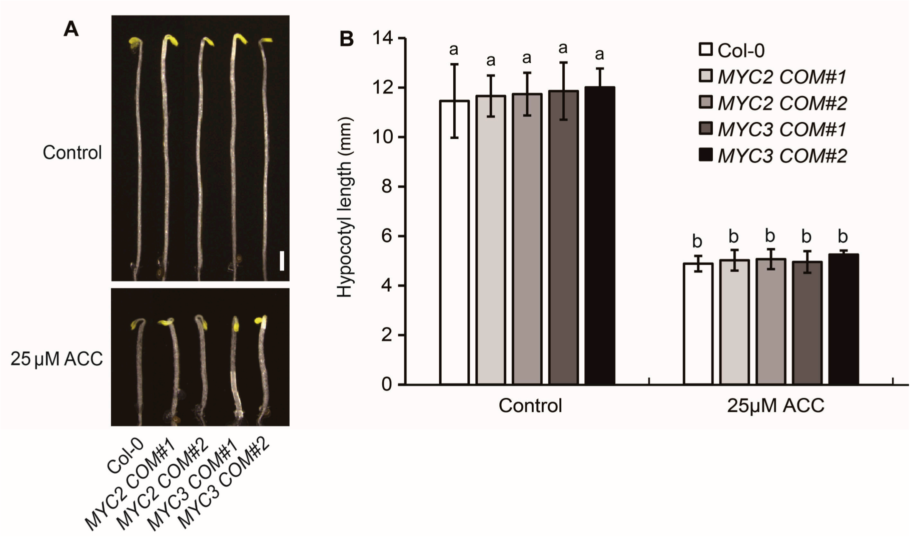

Figure S1. The effect of serial concentration of ACC on elongation of etiolated hypocotyls of wild-type and the complementation lines of *MYC2* or *MYC3*. (**A**). Photograph of etiolated seedlings which were grown on 1/2 MS medium containing 25 μM ACC for 4 days. Scale bar=1 mm. (**B**). Mean±SE of hypocotyl length. In each experiment, at least 40 seedlings were measured. Data from 3 replicates were calculated and statistically analyzed using one-way ANOVA.

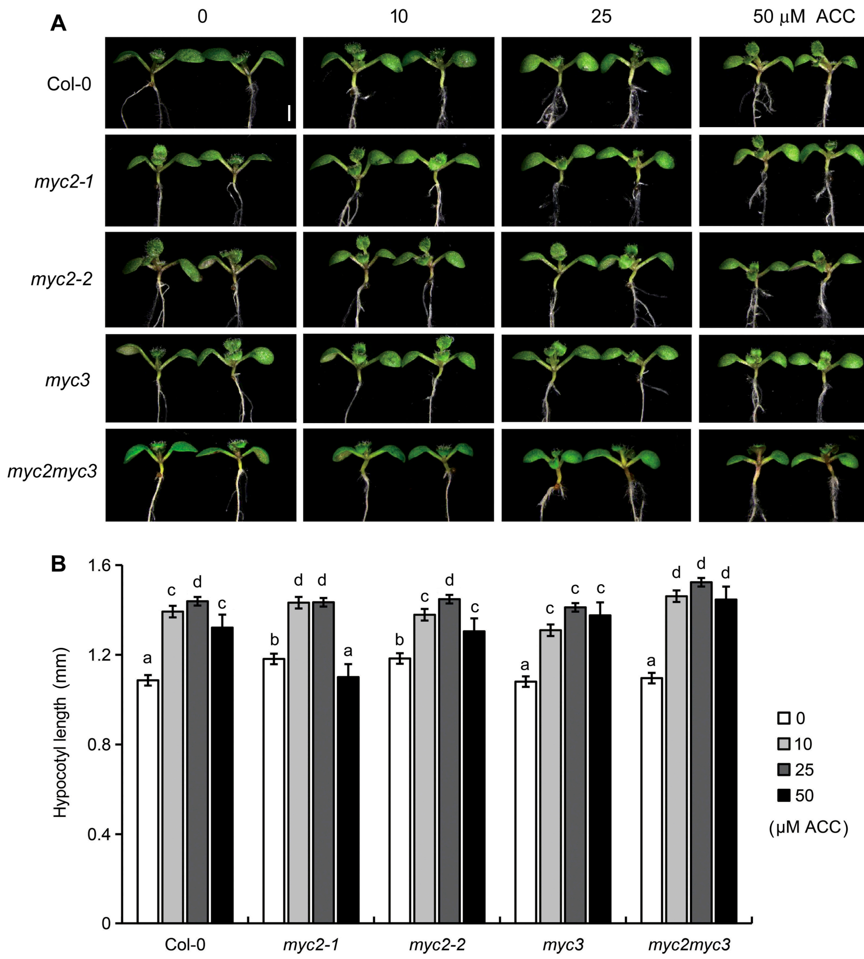

Figure S2. MYC2 or MYC3 is not involved in ethylene-promoted hypocotyl elongation of seedlings grown under light. (**A**). Photograph of seedlings that were grown on the serial concentration of ACC for 7 days. Scale bar=1 mm. (**B**). Mean±SE of hypocotyl length. In each experiment, at least 40 seedlings were measured. Data from 3 replicates were calculated and statistically analyzed using one-way ANOVA.

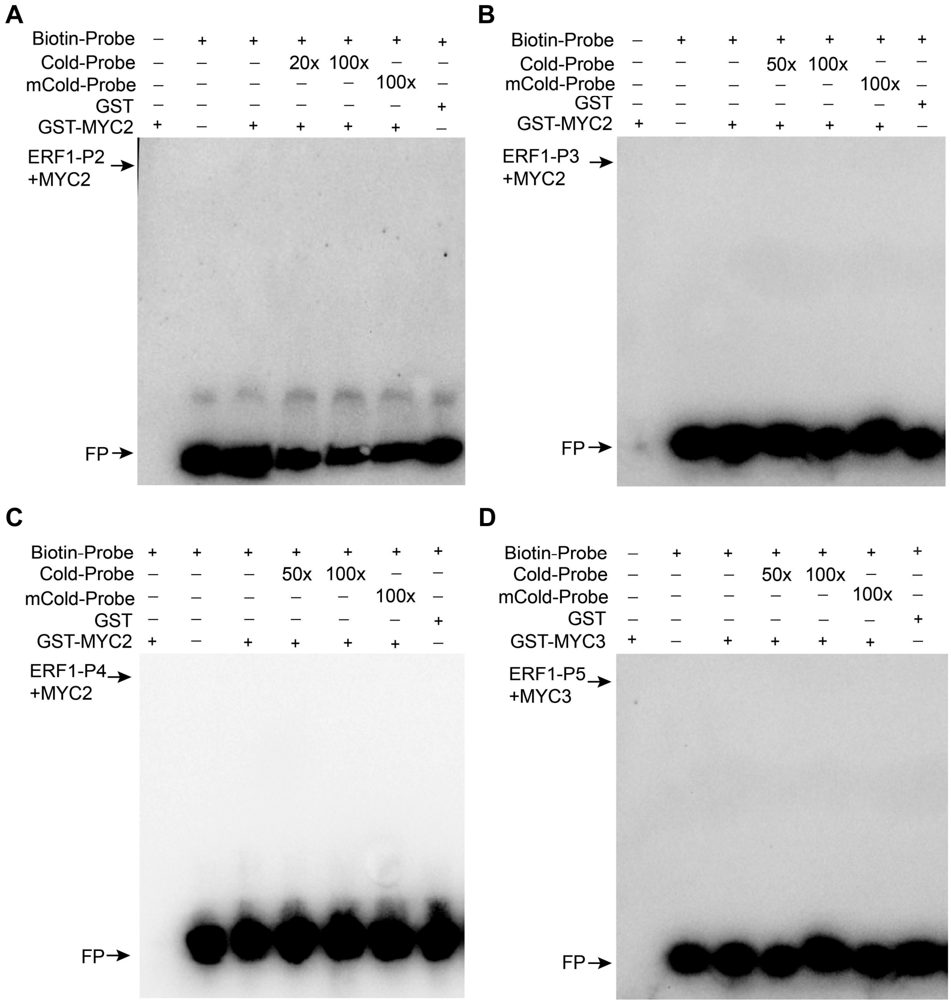

Figure S3. MYC2 or MYC3 did not bind P2-P5 of *ERF1* promoter. The binding of MYC2 or MYC3 with P2~P5 fragments in the *ERF1* promoter was detected using EMSA.

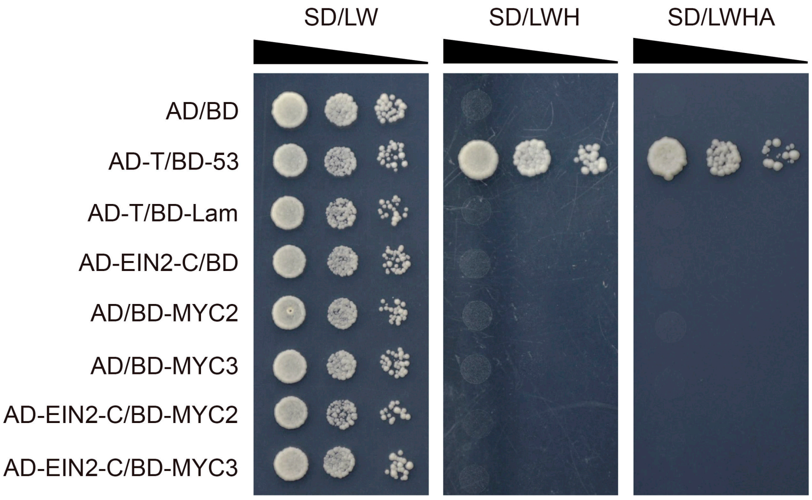

Figure S4. MYC2 or MYC3 did not interact with EIN2. Yeast two hybridization was used to detect the interaction between MYC2 or MYC3 with EIN2.

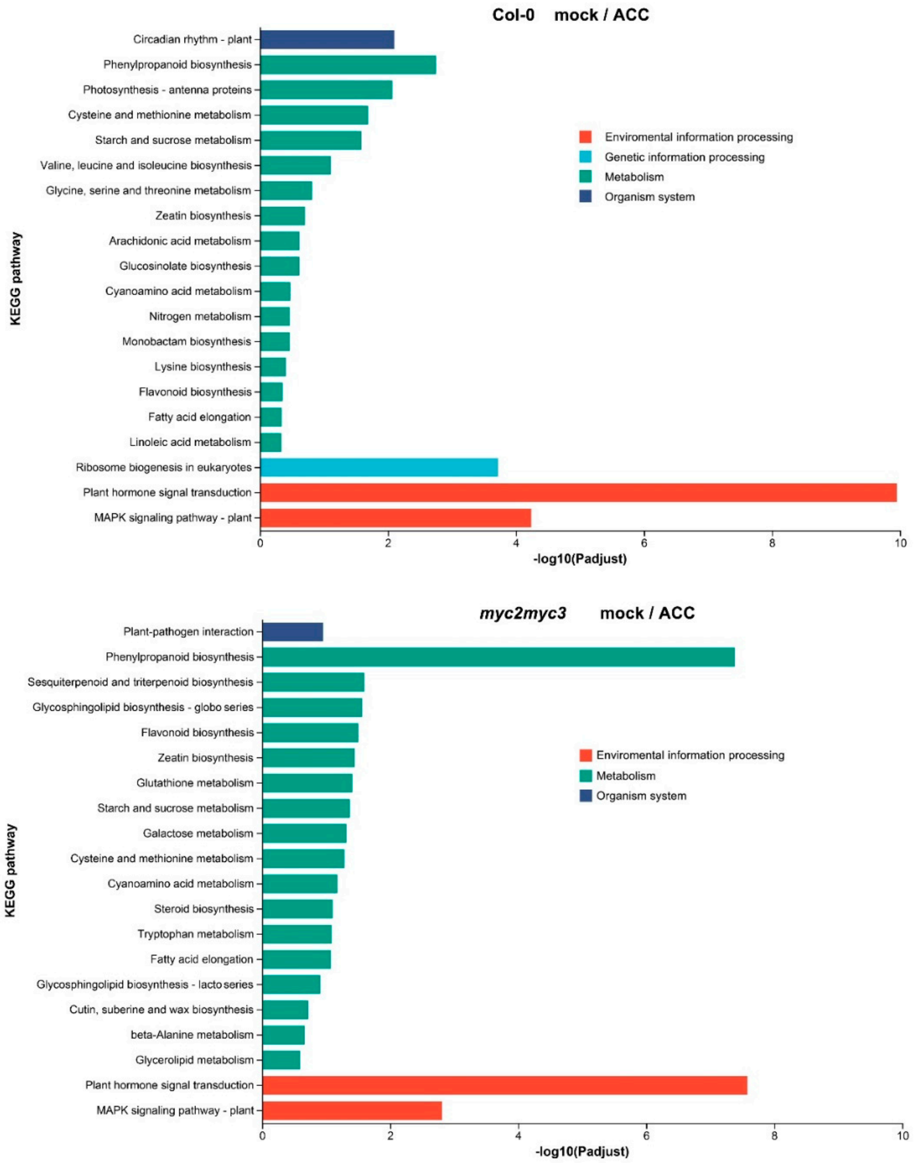

Figure S5. KEGG analysis of ethylene-regulated DEGs in Col-0 and *myc2myc3*. Etiolated seedlings were grown on a medium containing 25 μM ACC for 4 days. High-throughput RNA-seq was used to analyze the expression pattern of functional genes.

Table S1. DEGs that were up-regulated in *myc2myc3* while not up-regulated in Col-0 after ACC treatment

Functional category	Gene_id	Gene name	Gene description
cell wall organization	AT2G15345		plant invertase/pectin methylesterase inhibitor
	AT5G04970	PME47	plant invertase/pectin methylesterase inhibitor
	AT2G32620	CSLB2	Cellulose synthase-like protein B2
	AT2G43890		pectin lyase-like protein
	AT5G06630		proline-rich extensin-like protein
cellular metabolism	AT4G23900	NDK4	Nucleoside diphosphate kinase IV
	AT5G56080	NAS2	Nicotianamine synthase 2
	AT1G09240	NAS3	Nicotianamine synthase 3
	AT4G22880	LDOX	involved in proanthocyanin biosynthesis
	AT4G26500	SUFE1	involved in Fe-S cluster biogenesis in mitochondria and plastids
	AT2G42360	ATL41	E3 ubiquitin-protein ligase ATL41
	AT2G20160	ASK17	E3 ubiquitin ligase SCF complex subunit
	AT3G20520	GDPDL5	glycerophosphodiester phosphodiesterase
	AT2G02010	GAD4	involved in glutamate decarboxylation to succinate
cytoskeleton	AT5G07760		FH2 domain-containing protein, involved in actin organization
organization	AT4G19400		profilin family protein
signal transduction	AT4G09200		SPla/RYanodine receptor (SPRY) domain-containing protein
	AT1G76640	CML39	Calmodulin Like 39
	AT1G16090	WAKL7	Wall associated kinase-like 7
stress response	AT3G54430	SRS6	involved in stress response
	AT3G19710	BCAT4	involved in biosynthesis of glucosinolates
	AT5G26150		a protein kinase, involved in response to stress
transcription factor	AT1G65330	PHE1	MADS-box transcription factor
	AT3G17010	REM22	an AP2/B3-like transcriptional factor
	AT4G28800		bHLH transcription factor
	AT5G07310	ERF115	ethylene-responsive transcription factor
transport	AT1G06970	CHX14	a Na^+/H^+ antiporter
	AT1G06330		heavy metal transport/detoxification superfamily protein

Table S2. DEGs that were down-regulated in *myc2myc3* while not down-regulated in Col-0 after ACC treatment

Functional category	Gene_id	Gene name	Gene description
cellular metabolism	AT3G21370	BGLU19	Beta-glucosidase 19, involved in carbohydrate metabolism
	AT5G50580	SAE1B-2	SUMO-activating enzyme subunit 1B-1
	AT2G39850	SBT4.1	Subtilisin-like protease SBT4.1
	AT3G54940	RD19D	cysteine protease RD19D, involved in proteolysis
	AT1G14240	APY3	apyrase 3, involved in ATP hydrolysis
	AT5G59130	SBT4.11	Subtilisin-like protease, involved in proteolysis
	AT4G13280	TPS12	Terpenoid synthase 12,
	AT4G11310	RDL4	cysteine protease, involved in proteolysis
	AT2G28420	GLYI8	lactoylglutathione lyase/glyoxalase I family protein
	AT1G66800		involved in lignin biosynthesis
	AT2G05400		ubiquitin-specific protease
	AT1G33750	TPS22	Terpenoid synthase 22
growth & development	AT2G41260	M17	Late-embryogenesis-abundant (LEA) protein
	AT3G17520		LEA protein
	AT1G32560	LEA6	LEA protein
	AT3G53040		LEA protein
	AT1G22600		LEA protein
	AT2G28490		Vicilin-like seed storage protein
	AT5G42600	MRN1	oxidosqualene synthase that produces the monocyclic triterpene
	AT3G51810	EM1	ABA-inducible protein that accumulates during seed maturation
hormone metabolism or signaling	AT4G12550	AIR1	involved in response to auxin
	AT2G22810	ACS4	ACC synthase 4, involved in ethylene synthesis
	AT4G29740	CKX4	involved in degradation of cytokinin
	AT5G66400	RAB18	involved in ABA and ethylene signaling
	AT2G35990	LOG2	cytokinin riboside 5'-monophosphate phosphoribohydrolase
	AT1G78440	GA2OX1	involved in gibberellin inactivation
	AT1G12010		ACC oxidase 3
oxidation reduction	AT1G12130		Flavin-containing monooxygenase FMO GS-OX-like 6
	AT1G74110	CYP78A10	Cytochrome P450 family protein
	AT2G46950	CYP709B2	Cytochrome P450 family protein
	AT5G42590	CYP71A16	Cytochrome P450 family protein
	AT5G42580	CYP705A12	Cytochrome P450 family protein
	AT2G38390	PER23	Peroxidase 23
	AT5G50770	HSD6	11-β-hydroxysteroid dehydrogenase-like 6
signal transduction	AT5G10250	DOT3	phototropic-responsive NPH3 family protein
	AT2G02300	PP2B5	F-box protein PP2-B5
	AT3G05140	RBK2	a ROP binding protein kinase
stress response	AT5G38000		zinc-binding dehydrogenase, involved in response to oxidative stress
	AT1G19250	FMO1	flavin-dependent monooxygenase 1, promotes resistance to pathogen
	AT4G11210	DIR14	involved in lignan biosynthesis, and defense response
	AT1G66270	BGLU21	a beta-glucosidase, involved in response to salt, cold, and osmotic stress
transcription factor	AT5G24110	WRKY30	WRKY transcription factor
	AT4G28140	ERF054	Ethylene-responsive transcription factor 054
transport	AT5G38030	DTX30	DETOXIFICATION 30, involved in drug transmembrane transport
	AT2G04090	DTX5	DETOXIFICATION 5, involved in drug transmembrane transport
	AT5G59310	LTP4	non-specific lipid-transfer protein, up-regulated by ABA
	AT1G72125	NPF5.13	NRT1/ PTR FAMILY 5.13, involved in oligopeptide transport
